# The Role of Intravenous Tranexamic Acid for Posterior Spinal Fusion in Pediatric Non-Idiopathic Scoliosis: A Systematic Review and Meta-Analysis

**DOI:** 10.3390/jcm15166341

**Published:** 2026-08-17

**Authors:** Abdulrahman O. Al-Naseem, Yahya Ali, Latefah AlOtaibi, Adel Altarkait, Bisher Albisher, Asmaa Alkandari, Yousef Alkandari, Federico Cardahi, Salim Al Rawahi, Neil Saran, Jean A. Ouellet

**Affiliations:** 1 Division of Orthopaedic Surgery, Department of Surgery, McGill University, Montreal, QC H3A 0G4, Canada; 2 Department of Surgery, Jaber Al-Ahmad Hospital, Kuwait City 18338, Kuwait; 3 Department of Surgery, Al-Amiri Hospital, Kuwait City 15300, Kuwait; 4 Department of Surgery, Al-Jahra Hospital, Kuwait City 00040, Kuwait; 5 Department of Orthopedics, Khoula Hospital, Muscat 116, Oman

**Keywords:** TXA, tranexamic acid, non-idiopathic scoliosis, posterior spinal fusion

## Abstract

**Background:** Tranexamic acid (TXA) is increasingly used to reduce perioperative blood loss during scoliosis surgery; however, its effectiveness in pediatric patients with non-idiopathic scoliosis remains underexplored. Unlike adolescent idiopathic scoliosis, non-idiopathic scoliosis is frequently associated with neuromuscular, syndromic, or other underlying disorders that increase surgical complexity and may limit the generalizability of evidence from idiopathic populations. This systematic review and meta-analysis evaluated the impact of TXA on surgical outcomes in pediatric patients undergoing posterior spinal fusion for non-idiopathic scoliosis. **Methods:** This analysis encompassed five studies (comprising one randomized controlled trial and four observational studies) involving a total of 230 patients (TXA group: 98; control group: 132). The primary outcomes were estimated blood loss and the volume of packed red blood cells (PRBCs) transfused. Secondary outcomes included total transfusion amount, operation duration, complications, cell salvage application, preoperative hematocrit levels and blood loss percentage. A random-effects model was used. Mean difference (MD) was used for continuous outcomes, and odds ratio (OR) for dichotomous variables, both with 95% confidence intervals (CIs). **Results:** The use of TXA during posterior fusion surgery showed to be of benefit and was associated with significantly less estimated blood loss (*p* < 0.0001) and cell salvage volume transfusion (*p* < 0.0001). The remaining secondary outcomes did not show significant differences when compared with the control. **Conclusion:** Current evidence suggests that TXA is an effective blood conservation strategy in pediatric patients undergoing posterior spinal fusion for non-idiopathic scoliosis. However, the available evidence remains limited, particularly regarding perioperative complications, and further high-quality prospective studies are required to strengthen the evidence base. **Clinical relevance:** Exploring the use of TXA in scoliosis surgery has the potential to influence the standard of care, especially if associated with significantly more desirable post-operative and long-term outcomes.

## 1. Introduction

Scoliosis is one of the most common spinal deformities in children, and severe manifestations often necessitate posterior spinal fusion as a corrective surgical measure. While effective in halting curve progression and improving function, this procedure is associated with substantial perioperative blood loss and a notable dependence on allogeneic blood transfusions [1,2]. Among the various subtypes, children with neuromuscular or syndromic (non-idiopathic) scoliosis are more susceptible to intraoperative hemorrhage. This heightened risk is attributed to factors such as compromised vascular and muscular integrity, reduced coagulation reserve, and the need for extensive, multilevel spinal instrumentation [3,4].

To mitigate blood loss, various perioperative strategies have been employed, including cell salvage, controlled hypotension, and pharmacologic interventions [5]. Tranexamic acid (TXA) has been widely adopted for its ability to reduce surgical bleeding by inhibiting fibrinolysis via plasminogen binding [6,7]. TXA has demonstrated efficacy across a wide range of surgical domains, particularly in orthopedic and spine surgery [8,9].

In adolescent idiopathic scoliosis (AIS), multiple randomized and observational studies have shown that TXA significantly reduces intraoperative blood loss and transfusion requirements, with a favorable safety profile [10,11,12]. However, whether these findings are generalizable to patients with non-idiopathic scoliosis (NIS) remains uncertain. NIS encompasses a heterogeneous group of neuromuscular, syndromic, and other underlying disorders that are frequently associated with greater medical comorbidities, increased surgical complexity, and a higher risk of perioperative blood loss. Consequently, the external validity of evidence derived from AIS may be limited in this population, highlighting the need for a dedicated evaluation of TXA in NIS.

This systematic review and meta-analysis synthesizes the available evidence to evaluate the efficacy and safety of intravenously administered TXA in pediatric patients undergoing posterior spinal fusion for NIS. Specifically, this review assesses the effects of TXA on intraoperative blood loss, transfusion requirements, and perioperative complications to inform evidence-based blood management strategies in this high-risk population.

## 2. Materials and Methods

The systematic review and meta-analysis were conducted in accordance with the Preferred Reporting Items for Systematic Reviews and Meta-Analyses (PRISMA) guidelines [13]. PRISMA Checklist is in the Supplementary Material (Table S1).

### 2.1. Eligibility Criteria

Controlled trials and observational studies were included if they compared outcomes of scoliosis surgery with or without the use of intravenous tranexamic acid (TXA) for pediatric NIS (under the age of 18). Studies were excluded if they were not published in English, if they included adult participants (18 years or older), lacked a direct comparison between the two treatment groups, or missed key outcome data. The reference lists of all included studies were reviewed to identify additional relevant studies not captured in the initial search. Duplicate records were removed, and eligible studies were independently assessed by the research team for final inclusion.

### 2.2. Primary Outcomes

The primary outcomes were estimated intraoperative blood loss and volume of packed red blood cells transfused.

### 2.3. Secondary Outcomes

Secondary outcomes included preoperative hematocrit, total volume transfused, percentage of blood loss, volume of cell salvage used, operative time and total number of complications.

### 2.4. Literature Search Strategy

Two authors independently searched PubMed, EMBASE, and the Cochrane Central Register of Controlled Trials (CENTRAL), with the last search conducted on 9 July 2026. To identify unpublished or ongoing trials, the World Health Organization International Clinical Trials Registry and the ISRCTN Register were also searched. No language restrictions were applied.

### 2.5. Selection of Studies

Two authors independently screened the titles and abstracts of all identified studies. Full-text versions of relevant studies were retrieved, and those meeting the eligibility criteria were selected for final analysis. Discrepancies in study selection were resolved by discussion and consensus among the review team.

### 2.6. Data Extraction and Management

Data extraction followed the Cochrane data collection form for intervention reviews. A spreadsheet was pilot tested using randomly selected articles and refined accordingly. The data extraction sheet included:

Study characteristics: First author, year of publication, country of the corresponding author, journal, study design, sample size, clinical condition of participants, type of intervention, and comparator.

Baseline demographics: Age and gender of included participants.

Primary and secondary outcomes: Intraoperative blood loss, volume of packed red blood cells and other blood components transfused, pre- and postoperative laboratory results and reported complications.

Two authors independently extracted and recorded the data. Discrepancies were resolved through discussion until consensus was reached.

### 2.7. Data Analysis

Data were analyzed using Review Manager (RevMan) version 5.4. Two authors independently entered the data into the software. A random-effects model was used to calculate 95% confidence intervals (CIs). For dichotomous outcomes, the odds ratio (OR) was calculated, while the mean difference (MD) was used for continuous outcomes.

### 2.8. Assessment of Heterogeneity

Heterogeneity among studies was assessed using the Cochran Q test (χ^2^) and quantified with I^2^ statistics. The degree of heterogeneity was interpreted as follows:

0–25%: Low heterogeneity;

26–75%: Moderate heterogeneity;

76–100%: High heterogeneity.

### 2.9. Quality Assessment

The risk of bias in randomized controlled studies was assessed using the Cochrane Risk of Bias (ROB2) tool. For non-randomized studies, the Risk of Bias in Non-Randomized Studies of Interventions (ROBINS-I) tool was applied. Two authors independently conducted the quality assessment; any disagreements were resolved through discussion and consensus.

### 2.10. Sensitivity Analysis

A sensitivity analysis was conducted to evaluate the impact of individual studies on the overall results. Each study was sequentially excluded to assess its effect on the significance of the findings. The analysis confirmed that no single study, including those at high risk of bias, substantially altered the overall results.

## 3. Results

### 3.1. Literature Search Results

**Figure 1:**
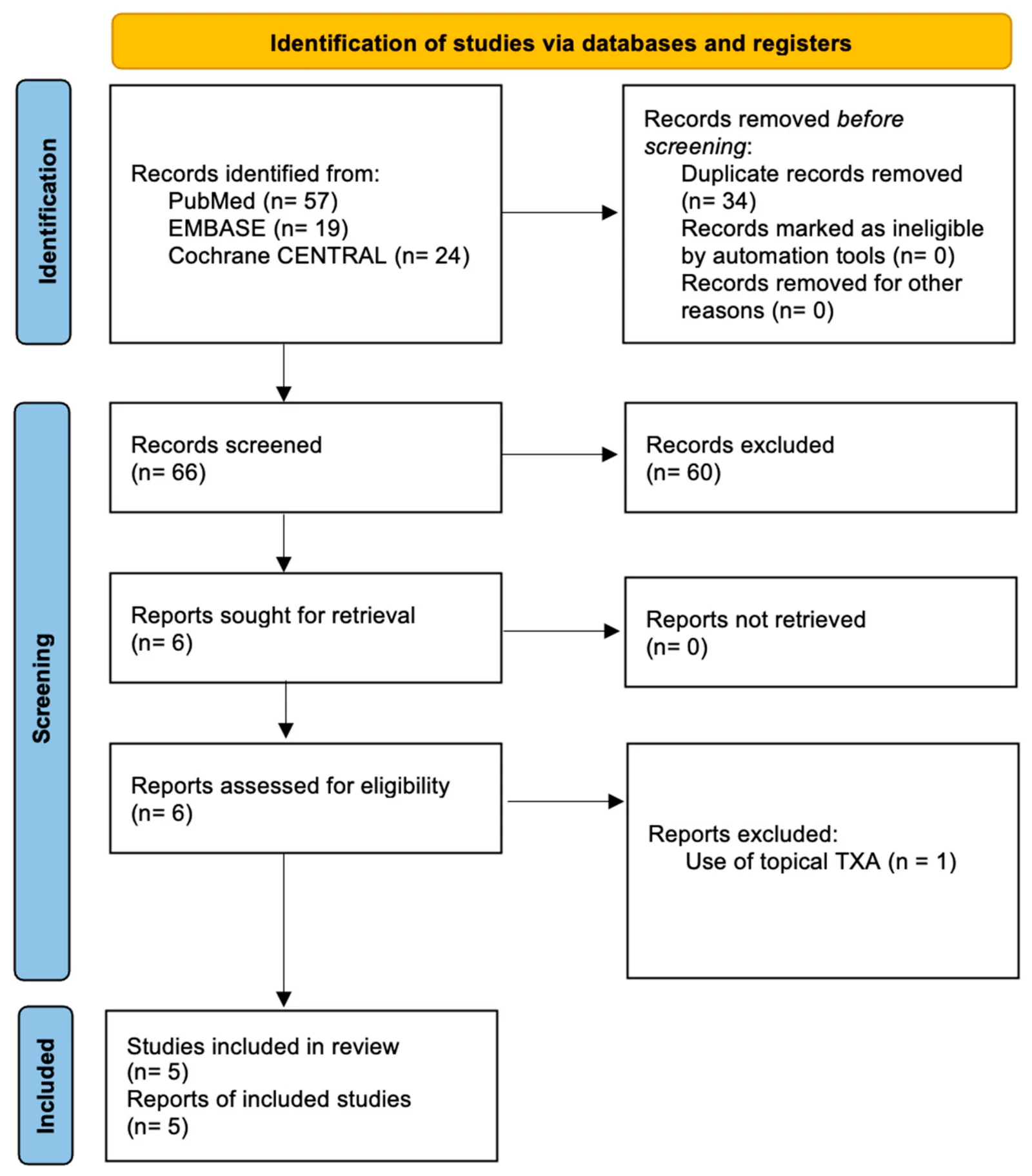
PRISMA Flow Diagram.

The literature search identified five studies. Following a thorough screening of the retrieved articles, all five studies met the eligibility criteria (Figure 1). These studies included a total of 230 patients, with 98 in the TXA group and 132 in the control group (Table 1).

### 3.2. Baseline Characteristics

A total of 230 patients were included in this review (TXA group: 98; control group: 132). The mean age of participants varied across studies, ranging from 12.70 ± 5.6 to 15.0 ± 3.4 years. Gender distribution was provided in most studies, with varying male-to-female ratios. The Cobb angles were reported in most studies, ranging from 45 ± 25.73 to 82 ± 20.7 degrees.

Table 1 summarizes the demographic characteristics of the patient populations in each study. Table 2 presents the etiology of secondary scoliosis among the included patients. Table 3 details intraoperative and postoperative outcomes, including operative time, intubation duration, number of vertebrae fused, ICU & hospital length of stay and complications. Table 4 presents at pre- and postoperative hematological parameters, including hematocrit and hemoglobin levels, as well as the transfused blood components and their volume.

### 3.3. Outcomes

#### 3.3.1. Total Volume Transfused (mL)

**Figure 2:**
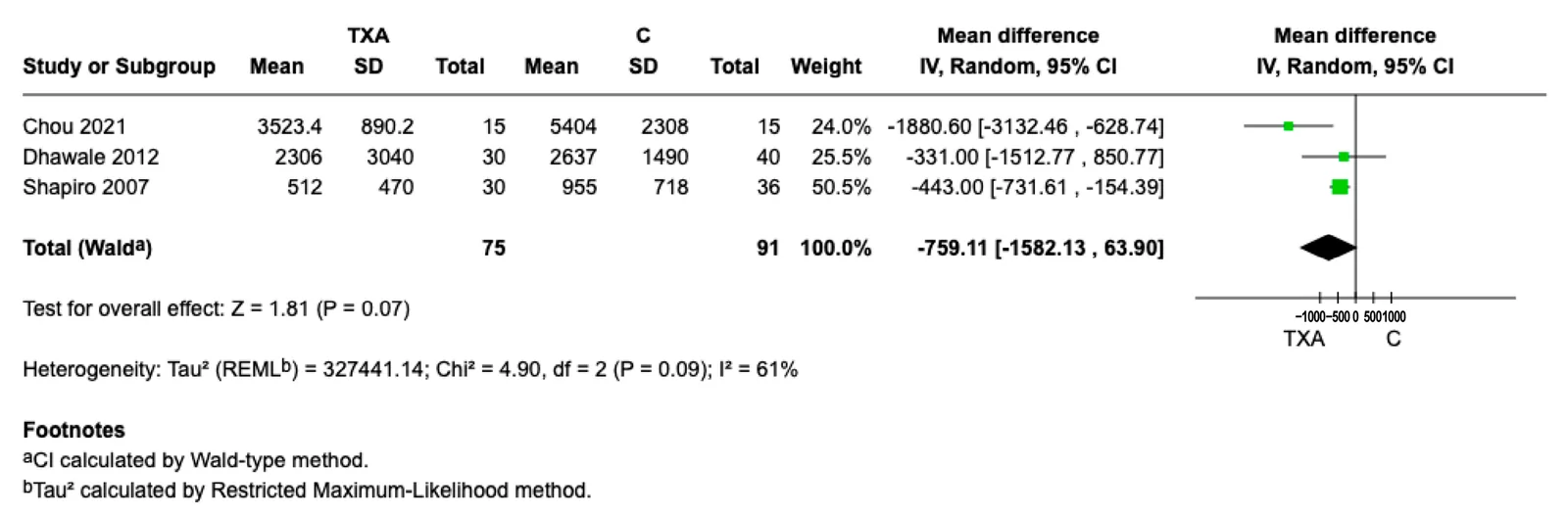
Total transfusion volume (mL) in TXA versus control [1,14,15].

Total volume transfused was reported in 3 studies, comprising 166 patients (Figure 2). There was no significant difference between TXA and control groups (MD = −759.11; 95% CI = −1582.25 to 63.90; *p* = 0.07). However, heterogeneity was moderate (I^2^ = 61%, *p* = 0.09).

#### 3.3.2. Total Estimated Blood Loss (mL)

**Figure 3:**
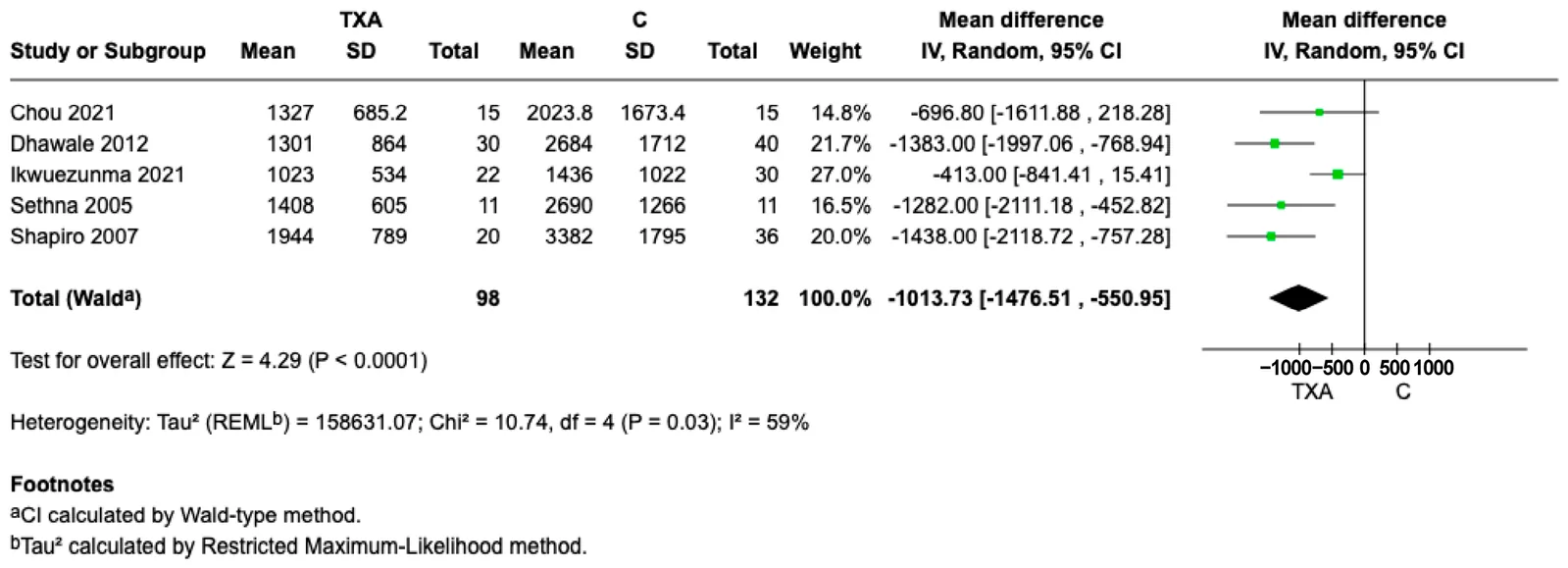
Estimated blood loss (mL) in TXA versus Control [1,10,15,16,17].

Estimated blood loss was reported in all five studies, comprising a total of 230 patients (Figure 3). TXA was associated with a significant reduction in blood loss compared with the control group (MD = −1013.73; 95% CI = −1476.51 to −550.95; *p* < 0.0001). A moderate degree of heterogeneity was present (I^2^ = 59%, *p* = 0.03).

#### 3.3.3. Cell Salvage (mL)

**Figure 4:**
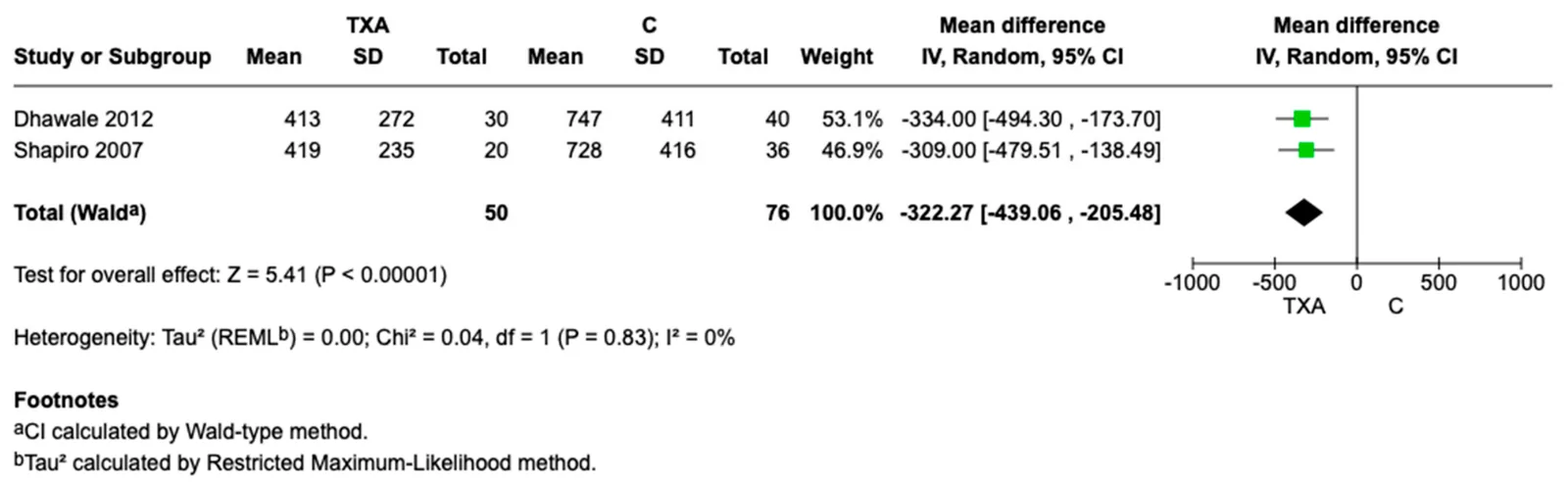
Cell salvage (mL) in TXA versus Control [1,14].

Volume of cell salvage used was reported in two studies, comprising a total of 126 patients (Figure 4). TXA was associated with lower cell salvage volumes when compared to the control group (MD = −322.27; 95% CI = −439.06 to −205.48; *p* < 0.00001). Heterogeneity was low (I^2^ = 0%, *p* = 0.83).

#### 3.3.4. All Complications

**Figure 5:**
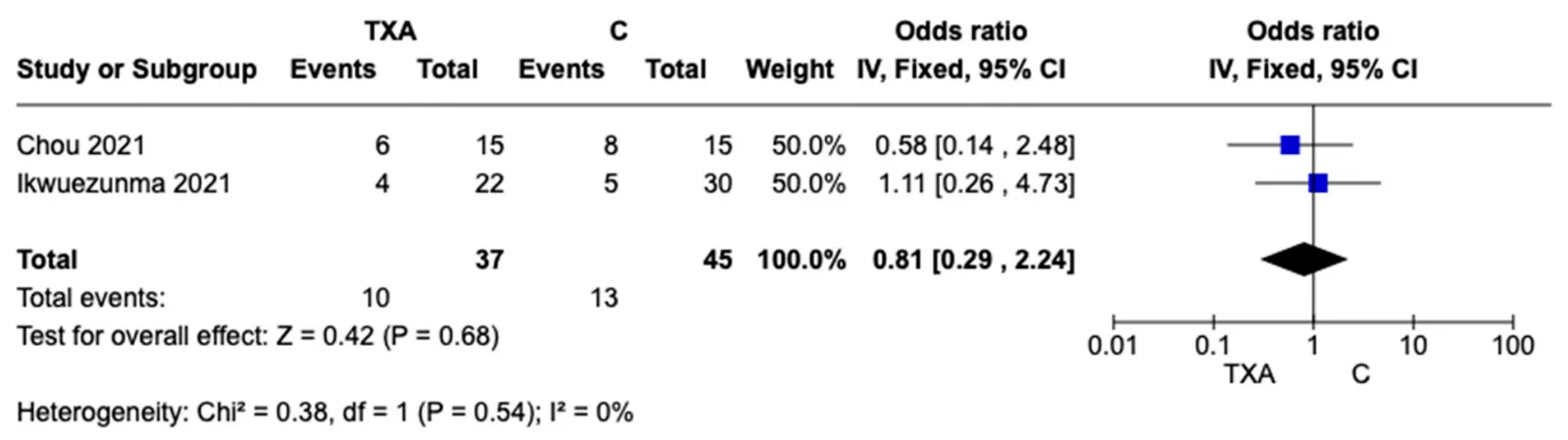
All complication rates in TXA versus Control [16,17].

Complication rates were reported in two studies, comprising a total of 82 patients (Figure 5). No significant difference was observed in complication rates between the TXA and control groups (OR = 0.81; 95% CI = 0.29 to 2.24; *p* = 0.68). Heterogeneity was very low (I^2^ = 0%, *p* = 0.54).

#### 3.3.5. Packed Red Blood Cells Transfused (mL)

**Figure 6:**
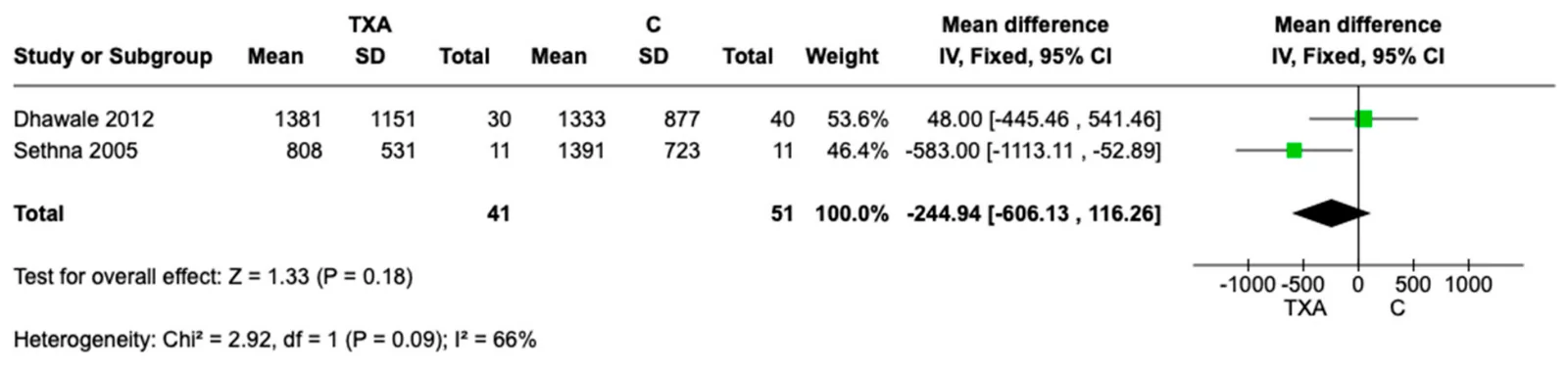
Packed red blood cells transfused (mL) in TXA versus Control [10,15].

The volume of packed red blood cells (PRBCs) transfused was reported in two studies, comprising a total of 92 patients (Figure 6). No significant difference was observed between both groups (MD = −244.94; 95% CI = −606.13 to 116.26; *p* = 0.18). Moderate heterogeneity was observed (I^2^ = 66%, *p* = 0.09).

#### 3.3.6. Operative Time (Minutes)

**Figure 7:**
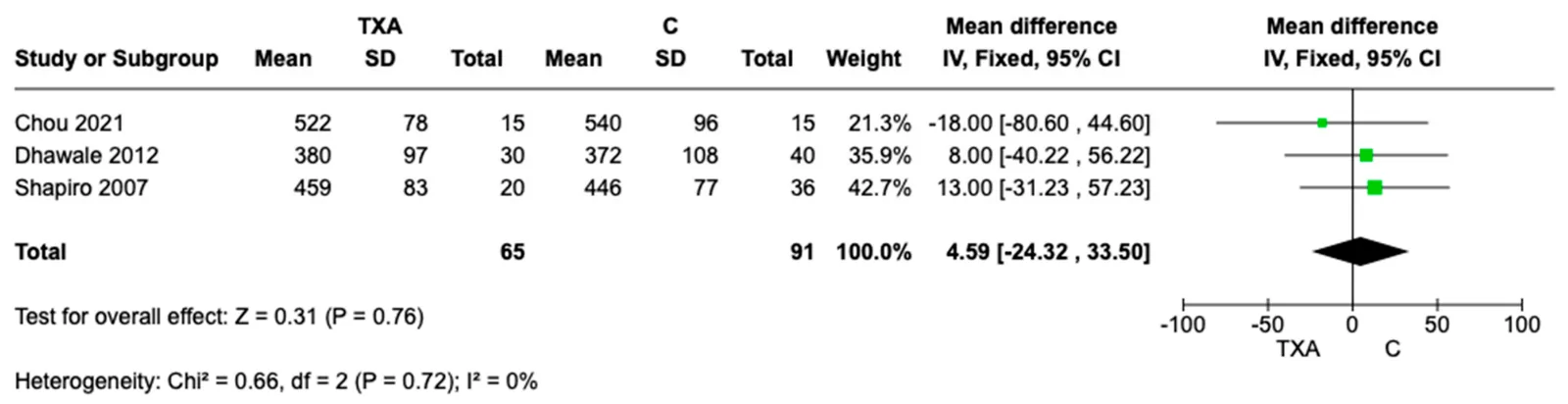
Operative time (minutes) in TXA versus Control [1,14,15].

Operative time was reported in three of the five studies, comprising a total of 156 patients (Figure 7). No significant result could be discerned between the TXA group and control group (MD = 4.59; 95% CI = −24.32 to 33.50; *p* = 0.76). A low degree of heterogeneity was observed (I^2^ = 0%, *p* = 0.72).

#### 3.3.7. Percentage of Blood Loss (%)

**Figure 8:**
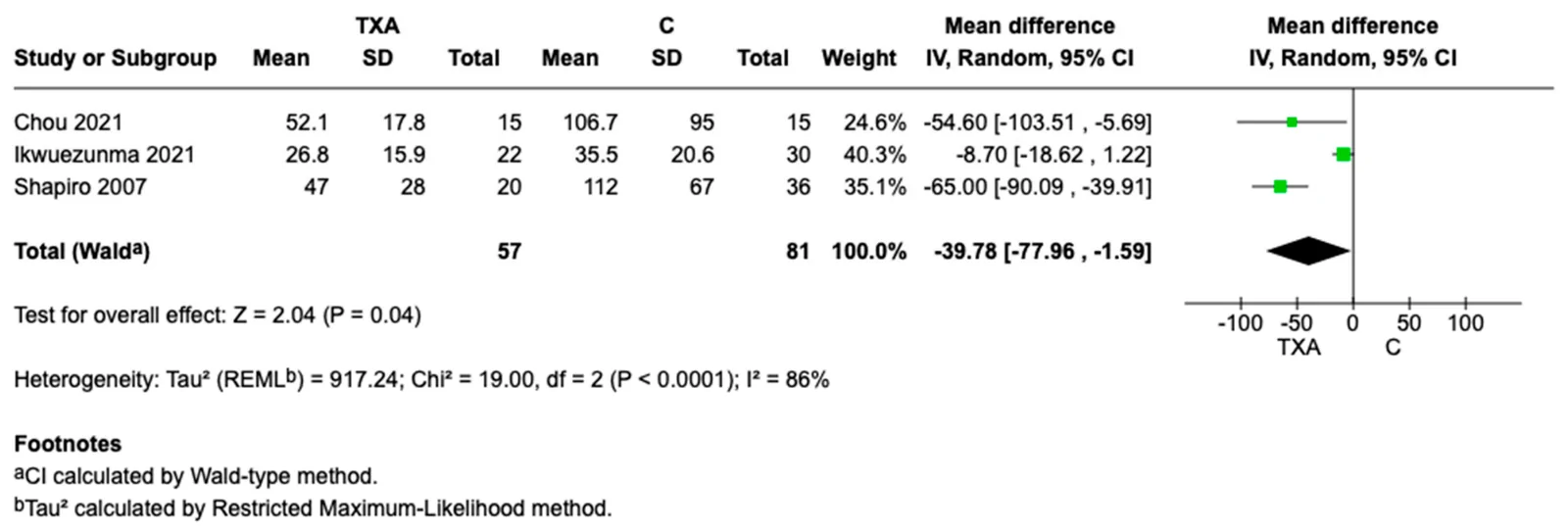
Percentage of blood loss (%) in TXA versus Control [1,16,17].

The percentage blood loss was reported in three studies, comprising a total of 138 patients (Figure 8). The results demonstrated a significantly lower percentage blood loss in the TXA group compared to the control (MD = −39.78; 95% CI = −77.96 to −1.59; *p* = 0.04). However, heterogeneity was substantially high, and this finding should therefore be interpreted with caution (I^2^ = 86%, *p* < 0.00001).

#### 3.3.8. Preoperative Hematocrit (%)

**Figure 9:**
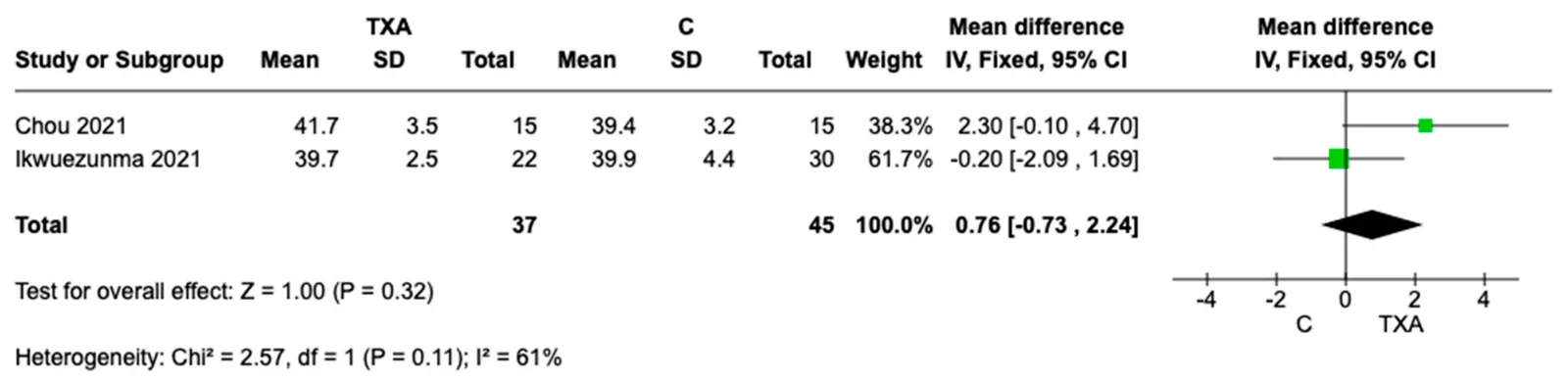
Preoperative hematocrit (%) in TXA versus Control [16,17].

The preoperative hematocrit was reported in two studies, comprising a total of 82 patients (Figure 9). No significant difference was observed between both groups (MD = 0.77; 95% CI = −0.73 to 2.24; *p* = 0.32). Heterogeneity was moderate (I^2^ = 61%, *p* = 0.11).

### 3.4. Quality Assessment Results

**Figure 10:**
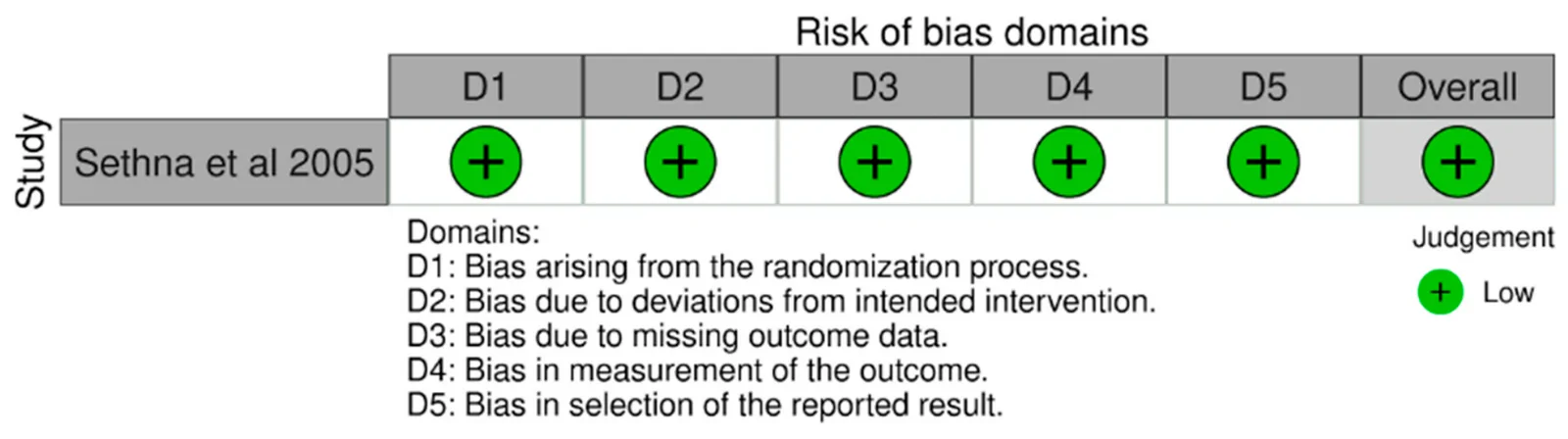
ROB2 risk of bias assessment for randomized studies [10].

**Figure 11:**
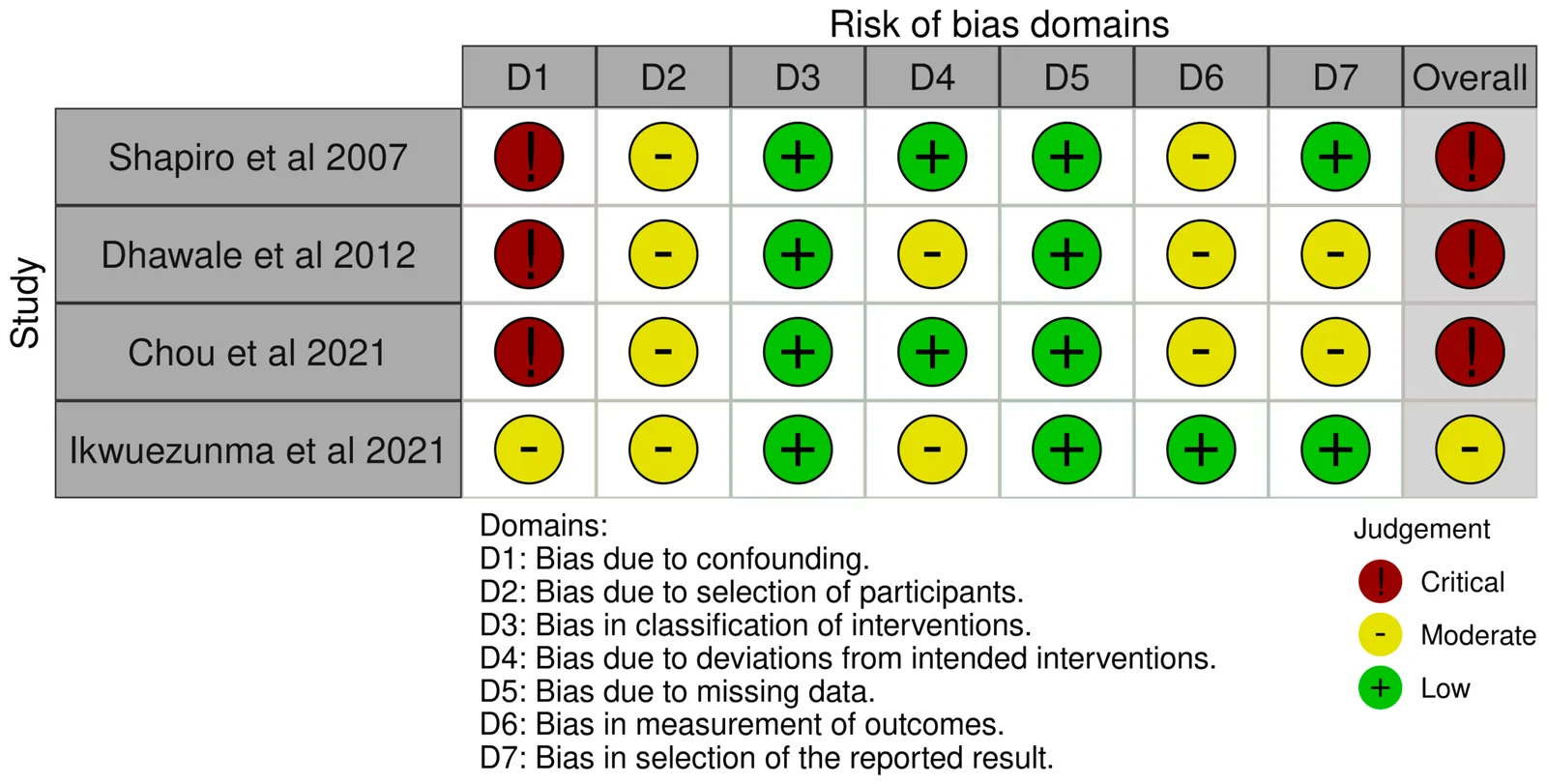
ROBINS-I risk of bias assessment for non-randomized studies [1,15,16,17].

The risk of bias in randomized controlled trials (RCTs) was assessed using the Cochrane Risk of Bias 2 (ROB 2) tool, while non-randomized studies were evaluated using the Cochrane (ROBINS-I) tool, as summarized in Figure 10 and Figure 11.

In ROB 2, Sethna et al. (2005) [10] demonstrated a low risk of bias, with no concerns identified in any domains. Among the five non-randomized studies assessed with ROBINS-I, three were judged to have a serious risk of bias, primarily due to concerns in the D1 domain, while the remaining domains showed low to moderate concerns. The other non-randomized study was rated as having a moderate risk of bias, with low to moderate concerns across the domains.

## 4. Discussion

This meta-analysis included five studies comprising 230 patients undergoing surgery for non-idiopathic scoliosis, with 98 receiving tranexamic acid (TXA) and 132 serving as controls. TXA was associated with significantly reduced estimated blood loss (EBL) and lower volumes of cell salvage and lower percentage blood loss. No significant differences were observed in total operative time, transfusion volume, packed red blood cell transfusions, pre-operative hematocrit levels and complication rates. Collectively, these findings suggest that TXA may improve perioperative blood conservation in patients with non-idiopathic scoliosis by reducing blood loss, while its effects on other perioperative outcomes remain uncertain.

TXA, an antifibrinolytic agent, has become integral to perioperative blood management because of its ability to stabilize fibrin clots and reduce surgical bleeding [7]. In adolescent idiopathic scoliosis (AIS), the evidence is extensive. Multiple randomized trials and meta-analyses confirm that TXA reduces blood loss and transfusion requirements, with pooled analyses showing decreases of approximately 700 mL in EBL and 250 mL in transfused volume [10,12]. A recent network meta-analysis suggested that higher-dose regimens (100 mg/kg bolus with 10 mg/kg/h infusion) are most effective in minimizing intraoperative bleeding and operative time [17]. Safety in AIS has also been well documented. Neilipovitz et al. reported no increase in seizures, thrombosis, or renal complications with TXA, while Goobie et al. observed no rise in thromboembolic events across pediatric cohorts [11,12]. These data support the efficacy and safety of TXA in AIS surgery.

In contrast, non-idiopathic scoliosis presents greater clinical complexity. Patients with neuromuscular or syndromic etiologies often have larger deformities, altered coagulation profiles, and longer fusion constructs, all of which contribute to substantial intraoperative bleeding and elevated morbidity [1,2,3]. Major blood loss can result in loss of neuromonitoring signals, risk of spinal cord injury, and postoperative anemia, which has been linked to delayed recovery and increased complications [1]. Blood transfusions themselves carry risks, including pulmonary compromise, coagulopathy, and infection, making blood conservation particularly important [2,5].

Our findings extend the existing evidence supporting TXA use in non-idiopathic scoliosis and are consistent with previous studies involving specific neuromuscular populations. Dhawale et al. reported lower EBL and transfusion needs in cerebral palsy patients, and Shapiro et al. demonstrated similar benefits in Duchenne muscular dystrophy [1,14]. More recent work by Chou et al. showed reductions in blood loss, transfusion requirements, and pulmonary complications in spinal muscular atrophy patients [15].

Although TXA has demonstrated consistent benefit, some theoretical risks remain. High-dose use has been linked to seizures in neurologically vulnerable patients, and thromboembolic events remain a concern in predisposed individuals. Nevertheless, most large-scale analyses have not demonstrated increased complication rates, supporting the continued evaluation of TXA as a potentially safe and effective adjunct, although evidence in non-idiopathic scoliosis remains limited [12].

Comparisons with other antifibrinolytics also underscore TXA’s advantage. Dhawale et al. demonstrated that TXA was more effective than epsilon-aminocaproic acid (EACA) and no antifibrinolytic use in children with cerebral palsy, significantly reducing EBL and cell salvage transfusion without added complications [14]. Mechanistically, TXA’s stronger binding affinity for plasminogen compared with EACA confers superior antifibrinolytic potency [7]. TXA has also supplanted aprotinin, which was historically used for its hemostatic effect but was withdrawn due to risks of anaphylaxis and renal toxicity, particularly in cardiac surgery [7]. In contrast, TXA appears to offer a favorable efficacy, safety, and cost profile across a variety of surgical disciplines, including spine surgery [8].

This study represents the first meta-analysis focused exclusively on TXA use in non-idiopathic pediatric scoliosis, a population historically underrepresented in antifibrinolytic research. The strengths of this study include the inclusion of diverse secondary scoliosis etiologies, which enhances external validity, and the assessment of multiple clinically meaningful outcomes, such as blood loss, transfusion and complication rates.

However, several limitations must be considered. The number of included studies and overall sample size remain modest, limiting statistical power and generalizability. Most included studies were non-randomized and at high or serious risk of bias, particularly in selection and outcome assessment. Substantial heterogeneity across dosing regimens, surgical techniques, and transfusion thresholds may have influenced pooled estimates; in particular, the pooled analysis for percentage blood loss demonstrated considerable statistical heterogeneity (I^2^ = 86%), likely reflecting differences in patient populations, surgical complexity, and TXA protocols. Accordingly, this result should be interpreted with caution. Furthermore, the included studies encompassed a heterogeneous group of non-idiopathic scoliosis etiologies, including cerebral palsy, Duchenne muscular dystrophy, spinal muscular atrophy, and Marfan syndrome. These conditions differ substantially in underlying pathophysiology, bleeding risk, comorbidities, and surgical complexity, which may have contributed to clinical heterogeneity and limits the generalizability of the pooled findings across all non-idiopathic scoliosis populations. Moreover, long-term outcomes and patient-reported measures were rarely reported, restricting the evaluation of TXA’s benefits beyond the immediate perioperative period.

Future research should prioritize prospective, multicenter randomized controlled trials (RCTs) with standardized TXA protocols tailored to non-idiopathic scoliosis populations. Optimal timing and dosing strategies warrant further exploration. Safety endpoints, including neuromonitoring changes and postoperative complications, should be consistently reported [1]. Finally, incorporation of patient-reported outcomes, quality-of-life measures, and cost-effectiveness analyses will be critical in establishing evidence-based perioperative blood management protocols for this vulnerable group.

## 5. Conclusions

This meta-analysis suggests that TXA may reduce blood loss and transfusion requirements in pediatric patients undergoing posterior spinal fusion for non-idiopathic scoliosis. Although the available evidence did not demonstrate an increased risk of perioperative complications or neuromonitoring alerts, these findings are based on a small number of predominantly observational studies and should therefore be interpreted with caution. Given the surgical complexity in this population, TXA may represent a valuable adjunct to comprehensive blood conservation strategies. Future multi-center randomized trials are warranted to better define establish the safety, efficacy and optimal use of TXA in this population.

## Figures and Tables

**Table 1:** Baseline characteristics of included studies.

Study ID	Sample Size		TXA Dose	Age ± SD		Sex (M/F)		Height (cm) ± SD		Weight (kg) ± SD		BMI (kg/m^2^) ± SD		Cobb Angle/Curvature (Degrees) ± SD	
	TXA	C	TX	TXA	C	TXA	C	TXA	C	TXA	C	TXA	C	TXA	C
Sethna et al. 2005 [10]	11	11	Loading dose 100 mg/kg TXA (100 mg/mL concentration), over 15 min. Infusion of TXA (10 mg/kg/h) continued until skin closure.	-	-	-	-	-	-	-	-	-	-	-	-
Shapiro et al. 2007 [1]	20	36	100 mg/kg IV over 15 min after induction of anesthesia and before incision. Infusion of 10 mg/kg per hour during surgery until skin closure.	13.9 ± 1.66	14.0 ± 2.06	20/0	36/0	-	-	-	-	-	-	51 ± 22.78	45 ± 25.73
Dhawale et al. 2012 [14]	30	40	TXA loading dose 100 mg/kg given for 20 to 30 min. 10 mg/kg Infusion until closure of the incision began.	-	14.7 ± 2.8	-	24/16	-	-	-	33.9 ± 10.6	-	-	76 ± 29.5	82 ± 20.7
Chou et al. 2021 [15]	15	15	IV loading dose of 100 mg/kg, followed by a maintenance dose of 10 mg/kg/h until skin closure.	12.7 ± 5.6	14.5 ± 5.5	5/10	6/9	150.1 ± 12.6	148.6 ± 14.2	39.6 ± 12.8	33.1 ± 8.6	-	-	70.3 ± 22.8	77.1 ± 29.1
Ikwuezunma et al. 2021 [16]	22	30	Either a 50-mg/kg loading dose with a 5-mg/kg/h continuous infusion or a 30 mg/kg loading dose with a 10 mg/kg/h continuous infusion.	14 ± 2.3	15 ± 3.4	7/15	12/18	-	-	56 ± 19	58 ± 17	-	-	70 ± 10	65 ± 15

TXA = Tranexamic Acid, C = Control.

**Table 2:** Causes of secondary scoliosis across studies.

Cause of Scoliosis	Study ID									
	Sethna et al. 2005 [10]		Shapiro et al. 2007 [1]		Dhawale et al. 2012 [14]		Chou et al. 2021 [15]		Ikwuezunma et al. 2021 [16]	
	TXA	C	TXA	C	TXA	C	TXA	C	TXA	C
Duchenne muscular dystrophy	6.0	8.0	20.0	36.0	-	-	-	-	-	-
Myelodyplasia	1.0	-	-	-	-	-	-	-	-	-
Central core myopathy	-	1.0	-	-	-	-	-	-	-	-
Mitochondrial disease	1.0	-	-	-	-	-	-	-	-	-
Cerebral palsy	1.0	-	-	-	30.0	40.0	-	-	-	-
Poliomyelitis	1.0	-	-	-	-	-	-	-	-	-
Angelman Syndrome	-	1.0	-	-	-	-	-	-	-	-
Emery–Dreifuss muscular dystrophy	1.0	-	-	-	-	-	-	-	-	-
Neurocutaneous disorders	-	1.0	-	-	-	-	-	-	-	-
Spinal Muscular Atrophy	-	-	-	-	-	-	15.0	15.0	-	-
Marfan’s Syndrome	-	-	-	-	-	-	-	-	22.0	30.0

**Table 3:** Perioperative data.

Study ID	Operative Time (min) ± SD		Intubation Duration (Hours) ± SD		No. of Vertebrae Fused ± SD		ICU Stay (Days) ± SD		Hospital Stay (Days) ± SD		Complications	
	TXA	C	TXA	C	TXA	C	TXA	C	TXA	C	TXA	C
Sethna et al. 2005 [10]	-	-	-	-	-	-	-	-	-	-	-	-
Shapiro et al. 2007 [1]	459.0 ± 83.0	446.0 ± 77.0	-	-	14.7 ± 0.8	14.3 ± 0.7	-	-	-	-	-	-
Dhawale et al. 2012 [14]	380.0 ± 97.0	372.0 ± 108.0	57.6± 84.0	91.2 ± 81.6	16.9 ± 0.6	16.7 ± 0.8	4.6 ± 4.6	5.7 ± 4.0	10 ± 6.5	14 ± 13.9	-	-
Chou et al. 2021 [15]	522.0 ± 78.0	540.0 ± 96.0	19.1 ± 12.9	52.8 ± 58.5	-	-	-	-	-	-	Wound infection: 1.0 Pneumonia + pulmonary edema: 2.0 Pulmonary edema: 2.0 Atelectasis: 1.0	Pneumonia + pulmonary edema: 6.0 Pulmonary edema: 1.0 Atelectasis: 1.0
Ikwuezunma et al. 2021 [16]	-	-	-	-	13.0 ± 2.0	13.0 ± 3.0	-	-	-	-	CSF leak: 3.0 Acute respiratory obstruction: 1.0	CSF leak: 4.0 Intraoperative CPR: 1.0

**Table 4:** Hematological parameters.

Study ID	Pre-Op Hb (g/dL) ± SD		Post-Op Day 1 Hb (g/dL) ± SD		Pre-Op Hematocrit (%) ± SD		Post-Op Day 1 Hematocrit (%) ± SD		Estimated Blood Loss (mL) ± SD		Estimated Blood Loss per Level (mL) ± SD		% Blood Loss ± SD		Total Volume Transfused (mL) ± SD		Packed Red Blood Cell Transfused (mL) ± SD		Platelet Volume Transfused (mL) ± SD	
	TXA	C	TXA	C	TXA	C	TXA	C	TXA	C	TXA	C	TXA	C	TXA	C	TXA	C	TXA	C
Sethna et al. 2005 [10]	-	-	-	-	-	-	-	-	1408.0 ± 605.0	2690.0 ± 1266.0	-	-	-	-	-	-	808.0 ± 531.0	1391.0 ± 723.0	-	-
Shapiro et al. 2007 [1]	-	-	-	-	-	-	-	-	1944.0 ± 789.0	3382.0 ± 1795.0	-	-	47.0 ± 28.0	112.0 ± 67.0	512.0 ± 470.0	955.0 ± 718.0	-	-	-	-
Dhawale et al. 2012 [14]	-	-	-	-	-	-	-	-	1301.0 ± 864.0	2684.0 ± 1712.0	77.0 ± 52.4	158.0 ± 98.5	-	-	2306.0 ± 3040.0	2637.0 ± 1490.0	1381.0 ± 1151.0	1333.0 ± 877.0	-	-
Chou et al. 2021 [15]	-	-	-	-	41.7 ± 3.5	39.4 ± 3.2	-	-	1327.0 ± 685.2	2023.8 ± 1673.4	-	-	52.1 ± 17.8	106.7 ± 95.0	3523.4 ± 890.2	5404.0 ± 2308.5	-	-	-	-
Ikwuezunma et al. 2021 [16]	13.1 ± 1.1	13.3 ± 1.5	-	-	39.7 ± 2.5	39.9 ± 4.4	-	-	1023.0 ± 534.0	1436.0 ± 1022.0	-	-	26.8 ± 15.9	35.5 ± 20.6	-	-	-	-	-	-

## Data Availability

No new data were created or analyzed in this study.

## Supplementary Materials

The following supporting information can be downloaded at: https://www.mdpi.com/article/10.3390/jcm15166341/s1, Table S1: PRISMA Checklist.
